# Effectiveness of a Peer-Led Pain Management Program in Relieving Chronic Pain and Enhancing Pain Self-Efficacy Among Older Adults: A Clustered Randomized Controlled Trial

**DOI:** 10.3389/fmed.2021.709141

**Published:** 2021-08-05

**Authors:** Mimi M. Y. Tse, Shamay S. M. Ng, Paul H. Lee, Xue Bai, Raymond Lo, Shuk Kwan Tang, Ka Long Chan, Yajie Li

**Affiliations:** ^1^School of Nursing, The Hong Kong Polytechnic University, Kowloon, Hong Kong; ^2^Department of Rehabilitation Sciences, The Hong Kong Polytechnic University, Kowloon, Hong Kong; ^3^Department of Health Sciences, The University of Leicester, Leicester, United Kingdom; ^4^Department of Applied Social Sciences, The Hong Kong Polytechnic University, Kowloon, Hong Kong; ^5^Department of Geriatrics and Palliative Medicine, Shatin Hospital, Hospital Authority, Ma On Shan, Hong Kong; ^6^Department of Land Surveying and Geo-Informatics, The Hong Kong Polytechnic University, Kowloon, Hong Kong; ^7^School of Nursing, University of Hong Kong, Hong Kong, Hong Kong

**Keywords:** chronic pain, nursing home residents, pain management program, peer-led, randomized controlled trial

## Abstract

Chronic pain is common in nursing home residents, who may have difficulty seeking out pain management strategies. Peer support model show promise as a strategy for managing chronic conditions. This was a clustered randomized controlled trial. A peer-led pain management program was provided for the experimental group. Pain situation, depression, quality of life, non-drug strategies used, and pain knowledge were measured. A total of 262 participants joined the study (146 were allocated as experimental group and 116 as control group). Before our intervention, the mean pain score reported was as high as 6.36 on a 10-point Likert Scale. The high intensity of their pain very much interfered with the daily activities of the participants. Pain interference was high and the participants had poor coping as indicated by the low pain self-efficacy. Depression and a low quality of life score was found. Upon completion of our PAP, there was a significant increase in pain self-efficacy, pain interference as well as quality of life for the participants in the experimental group and not in the control group, and this improvement sustained in 3-month follow up. The present study used a peer support models and proven to be effective in managing pain and pain related situations for nursing home residents with chronic pain. The peer volunteers involved in the pain management program taught relevant pain knowledge and pain management strategies to help our participants.

**Clinical Trial Registration:**https://clinicaltrials.gov/ct2/show/NCT03823495, NCT03823495.

## Introduction

Chronic non-cancer pain is a common condition among older adults, of which, will lead to big issues in physical and psychosocial health. Pain in older adults tends to be constant in nature, moderate to severe in intensity, and years long in duration (1–3). Older people with chronic pain are more anxious and more depressed as compared with their counterpart without pain (1–3).

Nursing home residents are physically frail and living in “closed” nursing home environments. It is alarming to find that majority of nursing home residents are suffering from chronic pain (2, 3). They may have difficulties in seeking help from health care professions in terms of pain management knowledge and strategies. Older adults often accept chronic pain as part of the process of aging, and have strong reservations in using oral medications as they afraid of possible adverse effects after taking pain relief drugs (4). It is therefore necessary to provide older adults with pain-related knowledge and relief strategies. In addition, the use of non-drug strategies for dealing with pain, including pain education, exercise, and visual stimulation is appealing.

We designed a chronic pain management program based on Ersek et al. (5) and Tse et al. (6). It includes pain management education, the use of oral drugs and non-drugs therapies, as well as various non-drug techniques (6). It has proven to be effective in reducing pain and improving the physical and psychological condition of nursing home residents. Given the limited resources in healthcare, healthcare professionals are encountering difficulties in providing adequate pain management in the caring process and the busy clinical settings (7–9). In this way, the use of peer support may be a good strategy.

Peer support had shown promising results in helping individuals to manager their chronic conditions (10, 11). Peer support involves “lay individuals with experiential knowledge who extend natural (embedded) social networks and complement professional health services” (12). Peer support can be illustrated in terms of providing emotional, informational and relationship support (10, 11). To examine the effectiveness of using peer support in managing pain for nursing home residents, we had conducted a pilot randomized controlled trial.

A total of 68 participants joined our pilot study (13), of which, thirty-six participants in the experimental group (with 12-week peer-led pain management program) and 32 participants in the control group (with usual care and a one page pain management pamphlet for self-reading). Pain self-efficacy and pain situations and characteristics had been improved in the experimental group as compared to the control group (13). In addition, peer volunteers suggested ways to improve the delivery of the pain management program to the older adults.

We had carried out our main study, a clustered randomized controlled trial, to explore the effectiveness of the pain management program (PAP) in nursing homes. In this paper, we report on the following: (1) a comparison of the pain self-efficacy, pain situations and characteristics of two groups, (2) examine the participants' quality of life experience and depression of two groups and (3) an exploration of the non-drug methods applied by the participants.

## Methods

### Study Design

This study presents the findings of a peer-led pain management program (PAP) that is a randomized controlled trial and registered on the ClinicalTrials.gov platform (NCT03823495). The study was conducted in nursing homes in Hong Kong. Ethical approval was obtained from The Hong Kong Polytechnic University and the participating nursing homes (Ref No. HSEARS20171218005).

### Sample and Procedure

Older adults were recruited from various government-subsidized nursing homes. The sample size was estimated based on the pain self-efficacy results in our pilot study. Using a significance level of 5% and a power of 80%, we arrived at a total estimated sample size of 256 participants with a medium effect size of 0.3789 (=6.1/16.1) on pain self-efficacy, taking into consideration a 10% drop-out rate (13) and a 0.1% intra-cluster correlation (from a review paper of 31 cluster-based studies in primary care) (14). Thus, 128 participants were needed for the experimental group and 128 for the control group. Eligible participants were recruited from Feb 2019 to Sept 2020. Data were collected in all groups at three time points: at baseline (T0), week 12 (T1, upon completion of the PAP) and week 24 (T2, to determine whether the observed benefits can be sustained over a longer period).

Inclusion criteria for nursing home residents:

i) aged >60 years.
ii) scored >6 in the Abbreviated Mental Test.
iii) experiencing non-cancer physical pain or discomfort either all the time or on and off for >3 months, with a pain score of ≥4 (on a 0–10-point pain scale in the Brief Pain Inventory.
iv) scored >60 in the Chinese version of the Modified Barthel Index.
v) Able to speak and understand Cantonese.

Exclusion criteria included:

i) had a history of psychotic disorders, making them unable to understand and follow instructions.
ii) had cancer and were currently undergoing cancer treatment.
iii) had a condition that limited them from safely participating in exercise (had a fracture or had recently undergone surgery, suffered an acute stroke, etc.

The research team sent letters and or email to invite nursing homes to join the PAP. Those nursing homes that expressed an interest in participating were randomized into either the experimental or control group using a computer-generated list, and, the participants (nursing homes) were not informed of the results. Nursing homes served as the unit of allocation, intervention, and analysis. Before starting the program, written informed consent was collected from all of the participants.

### Intervention

#### Pain Management Program (PAP) for the Experimental Group

A pain management program (PAP) led by peer volunteers (PVs) was provided for the participants in the experimental group. Details of the PAP were reported in our pilot study (13).

The PAP started with a 20 min physical exercises under the supervision of the PVs, then, 30 min pain management education about pain and the impacts of pain, the use of drugs and non-drug strategies, demonstrations and return demonstrations of non-drug pain management skills and techniques would be carried out by the PVs and nursing home residents.

Recruitment of the PVs: they were recruited from an institute hosted a university. Inclusion criteria of PVs were: (1) aged >55 year old; (2) scored over six in the Abbreviated Mental Test; (3) be willing to attend training workshops and meetings; (4) to demonstrate their ability to use various non-drug skills and techniques by passing an exit test; (5) be willing to lead the PAP in a nursing home. The training of the PVs included 8-h training workshops conducted over 2 weeks. Supplementary classes were given to those PVs that had missed out some of the training workshop.

The topics of the workshops: (1) communication skills; (2) client safety and confidentiality; (3) motivational strategies to enhance the compliance of the participants; (4) demonstrations on the use of the teaching manual (Appendix 1). PVs characteristics and their experience in leading the pain management program has been reported in a previous paper (15).

#### Control Group

The participants in the control group received the usual care and a pain management pamphlet distributed by the health care professions in the nursing homes, this is referencing to Ersek et al. (5). It is believed that reading the pamphlet are able to help those older adults to manage their pain situations, and that the efficacious will be lesser than the peer-led pain management program.

### Outcome Measures

Outcome measures included a series of well-designed, standardized questionnaires.

#### Pain Self-Efficacy

The Chinese version of Pain Self-Efficacy Questionnaire was used (16). Pain self-efficacy assess the participants' confidence in their ability to perform specific tasks or their confidence in performing more generalized constructs such as coping with chronic non-malignant pain. The pain self-efficacy questionnaire is a valid and reliable questionnaire. There were ten questions about patient's belief in his or her ability to accomplish daily tasks in spite of pain. The answers are rated on a 7-point scale, 0 = not at all confident, 6 = completely confident. The total score is calculated with a higher scores reflect greater pain-related self-efficacy (16). The Cronbach's alpha coefficient was 0.93 and the test-retest reliability was 0.75 (16).

#### Pain Intensity and Pain Interference

Pain intensity and pain interference were measured using the Brief Pain Inventory Chinese version (BPI-C) (17). There are four questions on pain severity and seven questions on pain interference. Items are rated from 0 = no pain to 10 = pain as bad as you can think of or interferes completely. Internal consistency and reliability were well-reported, with the Cronbach coefficient alpha was 0.894 and 0.915 respectively for pain intensity and pain interference (17).

#### Depression

The Chinese version of Geriatric Depression Scale (GDS) was used to measure depression. GDS has been tested and used extensively with the older population. Of the 15 items, 10 indicated the presence of depression when answered positively, while the rest (question numbers 1, 5, 7, 11, 13) indicated depression when answered negatively. The validity and reliability of the tool have been established, with the internal consistency (Cronbach α = 0.80) and test-retest reliability (r = 0.73) (18).

#### Perceived Quality of Life

The Chinese version of the SF-12 Questionnaire, which has 12 items derived from the physical and mental domains of the SF-36, was used. A separate summary of scores was obtained for each physical and mental domain by summing the scores across all 12 items. Higher scores indicate higher levels of health. All scores of the SF-12 showed good internal consistency (Cronbach's alpha 0.77–0.89).

#### Use of Non-drug Treatments

Information on the non-drug treatments, including listen to music, deep breathing, and exercises, that were used and the frequency (on a weekly basis) of usage were collected.

#### Pain Knowledge

To assess the participants' knowledge of pain management, a 11-item pain knowledge questionnaire was developed. Questions included: “Is exercise effective in pain management?”, “Can Paracetamol be used to treat fever and pain?”, “Is it appropriate to apply a hot or cold compress when sleeping?,” “Should deep breathing exercises be used to let the body relax before music therapy?.” The total score was calculated by counting the number of correctly answered questions, with high scores indicating better knowledge of pain.

The questionnaire were developed and validated by a panel. The Panel consisted a professor working in pain management, a geriatric physician consultant specializing in pain, a registered physiotherapist, and an advanced practice nurse with extensive experiences in caring of older adults.

### Statistical Analysis

Data were analyzed using SPSS version 25. Descriptive statistics and frequency distributions were calculated for sample characteristics. χ^2^ tests for categorical variables were used to evaluate for differences in demographic between the control and experimental groups. A *P-*value of < 0.05 was considered statistically significant.

Generalized estimating equations (GEE) with the identity link and first-order autoregressive [AR(1)] working correlation matrix were used to evaluate the effect of the intervention on primary (PSEQ) and secondary (BPI-Intensity and BPI-Interference, Pain knowledge, GDS, SF-12-Physical, SF-12-Mental, and usage of non-drug treatment) outcomes. GEE is an extension of generalized linear models that allows for the analysis of repeated measures with unknown covariance structure. GEE uses any and all available data that participants provide, even if follow-up data are missing (i.e., intent-to-treat analysis). For all models, the main effect of group (experiment group and control group) and time (baseline, post-intervention, and 3-month follow-up), and the Group × Time interaction were evaluated. Models were adjusted for sex, marital status, education, occupation.

Wald χ^2^ statistics with *P-*values < 0.05 for overall model effects were considered statistically significant. For models with significant Group × Time interactions, the main effects of group or time were not reported. GEE models for the entire study sample at all study time points (baseline, post-intervention, and 3-month follow-up) were evaluated.

Pairwise comparisons were used to examine the statistical significance Group by Time interactions observed in the GEE analyses to explore the difference in the outcomes between the experimental groups and control group at each follow-up time point. The alpha significance level for all analyses was set at 0.05. No adjustment was made to the alpha to compensate for the number of pairwise comparisons. To detect a meaningful clinical change, Cohen's d effect sizes and associated 95% confidence intervals were calculated for within-group and between-group based on the estimated marginal means derived from the GEE models.

## Results

### Demographic Results

**Figure 1:**
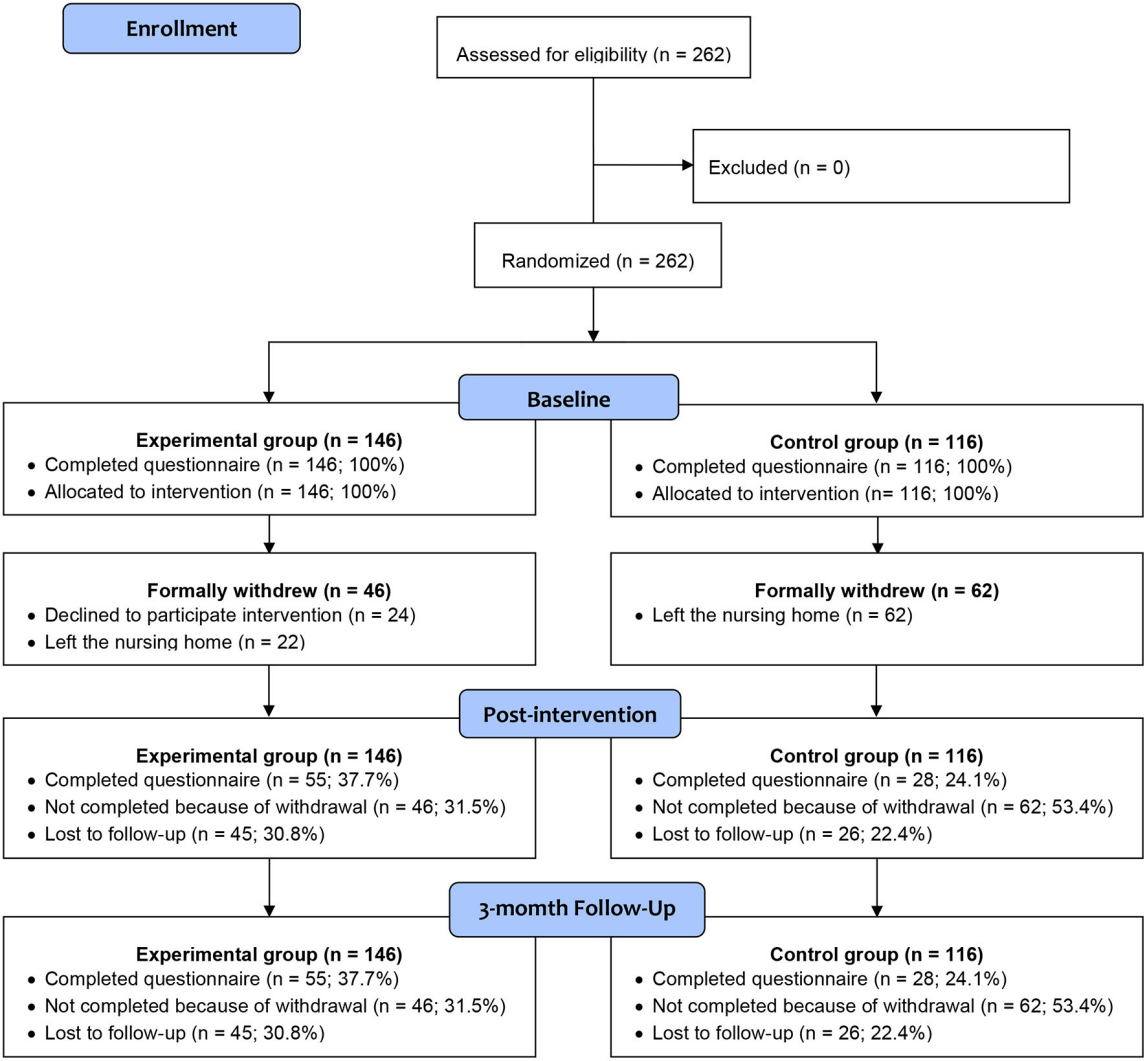
CONSORT 2010 flow diagram.

As shown in Figure 1, consort flow diagram, a total of 262 participants who satisfied the criteria were recruited. One hundred and forty-six participants were allocated to the experimental group and 116 to the control group. The demographic characteristics of the participants are shown in Table 1. More females (74.4%) joined the study than males (25.6%). Most of the participants were aged between 60 and 100. 68.3% of the participants were widows. More than one-third of the participants were uneducated. Nearly 40% of the participants had resided in a nursing home for 1–3 years, and more than 6% for over 10 years. Hypertension was the most commonly reported chronic disease (42.0%). No statistically significant differences in demographic characteristics were found between the experimental and control groups.

**Table 1:** Demographic characteristics of the experimental group and control group.

	**Total (*N* = 262)**	**Experimental group (*n* = 146)**	**Control group (*n* = 116)**	***p***
Gender, *N* (%)				0.216
F	195 (74.43)	113 (57.9)	82 (42.1)	
M	67 (25.57)	33 (49.3)	34 (50.7)	
Age group, *N* (%)				0.396
<60	3 (1.1)	1 (33.3)	2 (66.7)	
60–70	7 (2.7)	3 (42.9)	4 (57.1)	
71–80	48 (18.3)	23 (47.9)	25 (52.1)	
81–90	136 (51.9)	75 (55.1)	61 (44.9)	
91–100	65 (24.8)	42 (64.6)	23 (35.4)	
>100	1 (0.4)	1 (100.0)	0	
Marital status, *N* (%)				0.256
Single	14 (5.3)	7 (50.0)	7 (50.0)	
Married	47 (17.9)	28 (59.6)	19 (40.4)	
Divorced	20 (7.6)	7 (35.0)	13 (65.0)	
Widowed	179 (68.3)	102 (57.0)	77 (43.0)	
Educational level, *N* (%)				0.889
Uneducated	111 (42.4)	63 (56.8)	48 (43.2)	
Primary school	105 (40.1)	56 (53.3)	49 (46.7)	
Secondary school	40 (15.3)	23 (57.5)	17 (42.5)	
University or above	6 (2.3)	4 (66.7)	2 (33.3)	
Occupation, *N* (%)				0.999
Physical labor	119 (45.4)	67 (56.3)	52 (43.7)	
Technical job	74 (28.2)	41 (55.4)	33 (44.6)	
Housewife	30 (11.5)	17 (56.7)	13 (43.3)	
Clerk	19 (7.3)	11 (57.9)	8 (42.1)	
Others	17 (6.5)	10 (58.8)	7 (41.2)	
Nursing home staying, *N* (%)				0.657
<1 year	51 (19.5)	30 (58.8)	21 (41.2)	
1–3 years	103 (39.3)	54 (52.4)	49 (47.6)	
4–5 years	38 (14.5)	18 (47.4)	20 (52.6)	
6–10 years	34 (13)	21 (61.8)	13 (38.2)	
>10 years	16 (6.1)	10 (62.5)	6 (37.5)	
Others	1 (0.4)	1 (100.0)	0	
Chronic diseases, *N* (%)				
Hypertension	110 (42)	58 (52.7)	52 (47.3)	0.460
Cataract	73 (27.9)	41 (56.2)	32 (43.8)	0.874
Diabetes	65 (24.8)	35 (53.8)	30 (46.2)	0.773
Heart disease	45 (17.2)	25 (55.6)	20 (44.4)	0.980
Arthritis	39 (14.9)	23 (59.0)	16 (41.0)	0.625
Stroke	28 (10.7)	17 (60.7)	11 (39.3)	0.548
Other chronic disease	23 (8.8)	10 (43.5)	13 (56.5)	0.229
Tracheal disease	9 (3.4)	5 (55.6)	4 (44.4)	0.992
Parkinson disease	4 (1.5)	2 (50.0)	2 (50.0)	0.827
Physical disability	4 (1.5)	1 (25.0)	3 (75.0)	0.218

### Pain Self-Efficacy

**Figure 2:**
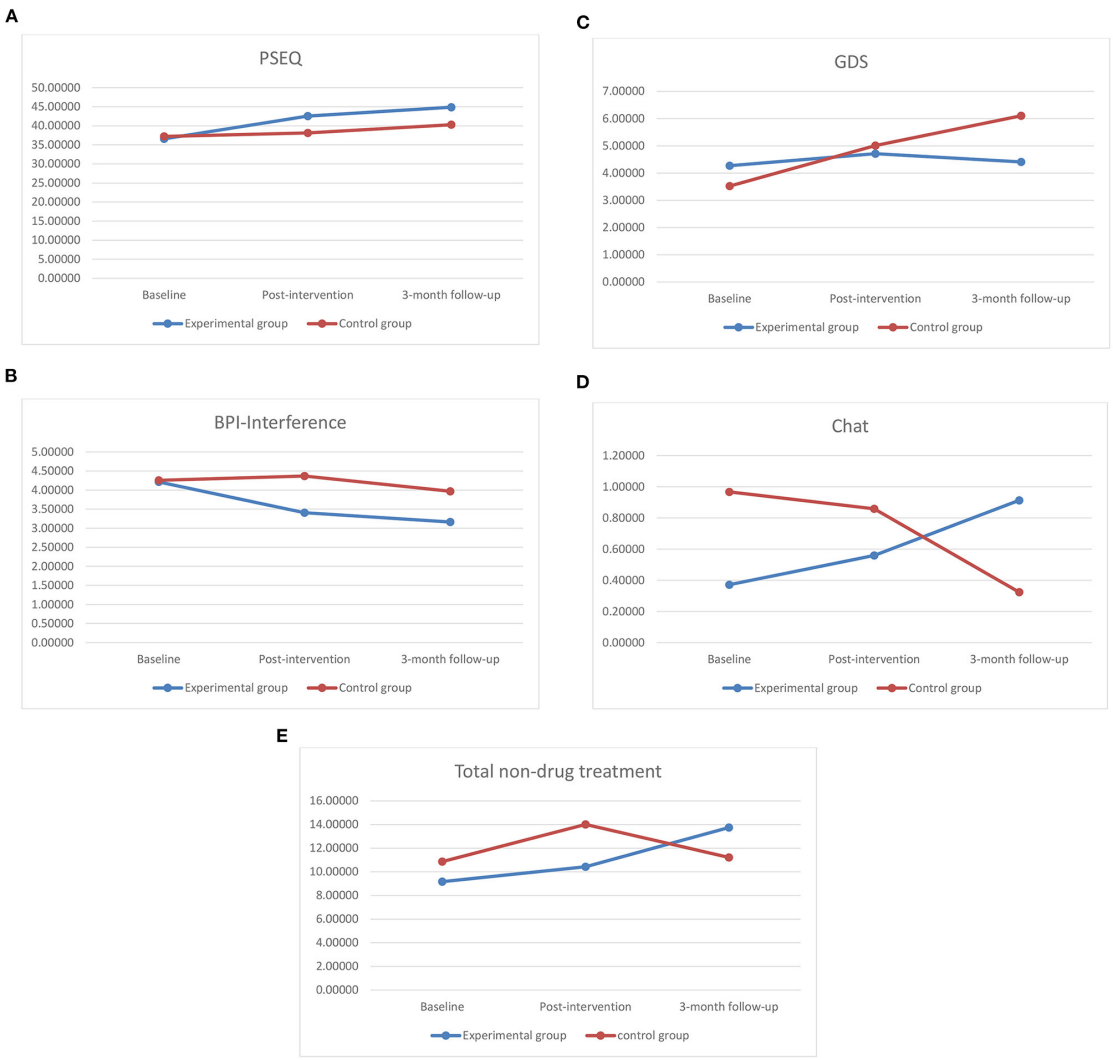
Estimated marginal means plotted at each measurement time point for GEE models that showed significant Group × Time interactions. Change over time in **(A)** PSEQ, **(B)** BPI-Interference, **(C)** GDS, **(D)** Chat, and **(E)** Total non-drug treatment.

In Figure 2, Tables 2, 3, the GEE analyses revealed significant Time X Group interactions were observed on the PSEQ (Wald's χ^2^ = 7.52, *P* = 0.023). Within-group comparison revealed statistically significant increases from baseline to post-intervention on PSEQ (*p* < 0.001) for the experimental group, while no significant difference was found for the control group (*p* = 0.568). Importantly, at post-intervention, between-comparison revealed that the experimental group had higher scores compared to the control group on the PSEQ (*p* = 0.023). In terms of follow-up, for the experimental group, there were no differences between post-intervention and 3-month follow-up scores for the experimental group (*p* = 0.152) and the control group (*p* = 0.361). This indicate that the improvement in post-intervention would be sustain in 3-month follow-up.

**Table 2A:** Test of model effects using GEE for primary and secondary outcomes.

	**WALD **χ**^**2**^**	**df**	***p***
**PSEQ**			
Time	22.073	2	0.000
Group	3.926	1	0.048
Time × Group interaction	7.515	2	0.023
**BPI-interference**			
Time	7.037	2	0.030
Group	4.425	1	0.035
Time × Group interaction	6.501	2	0.039
**BPI-intensity**			
Time	6.394	2	0.041
Group	2.745	1	0.098
Time × Group interaction	2.580	2	0.275
**Pain knowledge**			
Time	9.815	2	0.007
Group	0.204	1	0.652
Time × Group interaction	0.444	2	0.801
**GDS**			
Time	16.170	2	0.000
Group	0.882	1	0.348
Time × Group interaction	10.504	2	0.005
**SF-12-physical**			
Time	5.156	2	0.076
Group	0.036	1	0.849
Time × Group interaction	0.092	2	0.955
**SF-12-mental**			
Time	1.363	2	0.506
Group	0.027	1	0.870
Time × Group interaction	0.872	2	0.646

*PSEQ, Pain Self-Efficacy Questionnaire; BPI, Brief Pain Inventory; GDS, Geriatric Depression Scale*.

**Table 2B:** Test of model effects using GEE for non-drug treatment.

	**WALD **χ**^**2**^**	**df**	***p***
**Hot compress**			
Time	2.203	2	0.332
Group	2.496	1	0.114
Time × Group interaction	4.246	2	0.120
**Cold compress**			
Time	1.082	2	0.582
Group	3.045	1	0.081
Time × Group interaction	3.531	2	0.171
**Massage**			
Time	7.826	2	0.020
Group	0.025	1	0.875
Time × Group interaction	3.052	2	0.217
**Rest**			
Time	15.587	2	0.000
Group	0.556	1	0.456
Time × Group interaction	1.781	2	0.411
**Listen to music**			
Time	5.049	2	0.080
Group	0.613	1	0.434
Time × Group interaction	1.832	2	0.400
**Watch TV**			
Time	11.700	2	0.003
Group	5.786	1	0.016
Time × Group interaction	0.688	2	0.709
**Chat**			
Time	0.127	2	0.938
Group	0.289	1	0.591
Time × Group interaction	6.213	2	0.045
**Meditation**			
Time	2.102	2	0.350
Group	0.329	1	0.566
Time × Group interaction	2.020	2	0.364
**Breath**			
Time	6.115	2	0.047
Group	0.135	1	0.713
Time × Group interaction	2.757	2	0.252
**Exercise**			
Time	16.807	2	0.000
Group	1.178	1	0.278
Time × Group interaction	1.546	2	0.462
**Total non-drug treatment**			
Time	12.121	2	0.002
Group	1.109	1	0.292
Time × Group interaction	7.294	2	0.026

**Table 3:** Estimated marginal means and standard deviation at each measurement time point for GEE models that showed significant Group × Time interaction.

	**Estimated marginal mean**		**Within-group** ***p*** **-value** ^**a**^		**Between-group *p*-value**
	**Experimental group**	**Control group**	**Experimental group**	**Control group**	
**PSEQ**					
Baseline	36.57 ± 27.93	37.21 ± 23.50			0.683
Post-intervention	42.58 ± 29.93	38.13 ± 26.63	<0.001	0.568	0.023
3-month follow-up	44.88 ± 30.15	40.28 ± 33.89	0.152	0.361	0.070
**BPI-interference**					
Baseline	4.21 ± 5.50	4.25 ± 4.32			0.887
Post-intervention	3.41 ± 5.44	4.37 ± 5.08	0.001	0.676	0.009
3-month follow-up	3.16 ± 5.29	3.97 ± 7.35	0.351	0.368	0.112
**GDS**					
Baseline	4.27 ± 9.48	3.52 ± 7.12			0.078
Post-intervention	4.71 ± 9.85	5.01 ± 9.06	0.226	0.007	0.652
3-month follow-up	4.41 ± 10.8	6.1 ± 10.33	0.535	0.123	0.025
**Chat**					
Baseline	0.37 ± 3.58	0.97 ± 4.22			0.022
Post-intervention	0.56 ± 4.07	0.86 ± 4.41	0.385	0.777	0.361
3-month follow-up	0.91 ± 5.36	0.32 ± 4.74	0.297	0.157	0.140
**Total non-drug treatment**					
Baseline	9.17 ± 22.65	10.87 ± 20.41			0.061
Post-intervention	10.44 ± 24.54	14.01 ± 24.93	0.154	0.018	0.009
3-month follow-up	13.75 ± 28.81	11.22 ± 26.33	0.009	0.144	0.167

a *Baseline vs post-intervention, and post-intervention vs 3-month follow-up*.

*PSEQ, Pain Self-Efficacy Questionnaire; BPI, Brief Pain Inventory; GDS, Geriatric Depression Scale*.

Within- and between-group effect sizes from the GEE models are shown in Table 4. Relative to the baseline, small within-group effect sizes (Cohen's d) were observed for the PSEQ at post-intervention (*d* = −0.21) and 3-month follow-up (*d* = −0.29) compared to minimal effect sizes for control group for the PSEQ at post-intervention (*d* = −0.04) and 3-month follow-up (*d* = −0.11). For the experimental group relative to the control group, small between-group effect sizes (Cohen's d) were observed for the at post-intervention (*d* = 0.16) and at 3-month follow-up (*d* = 0.15).

**Table 4:** Effect size at each measurement time point for GEE models that showed significant Group × Time interaction.

	**Within-group effect size from baseline**		**Between-group effect size**
	**Experimental group**	**Control group**	
**PSEQ**			
Baseline			−0.02 (−0.21 to 0.16)
Post-intervention	−0.21 (−0.37 to −0.05)	−0.04 (−0.24 to 0.16)	0.16 (−0.03 to 0.34)
3-month follow-up	−0.29 (−0.45 to −0.12)	−0.11 (−0.31 to 0.10)	0.15 (−0.04 to 0.33)
**BPI-Interference**			
Baseline			−0.01 (−0.19 to 0.17)
Post-intervention	0.15 (−0.01 to 0.31)	−0.02 (−0.22 to 0.18)	−0.18 (−0.36 to 0.00)
3-month follow-up	0.20 (0.04 to 0.35)	0.05 (-0.15 to 0.25)	−0.13 (−0.31 to 0.05)
**GDS**			
Baseline			0.09 (−0.10 to 0.27)
Post-intervention	−0.05 (−0.21 to 0.11)	−0.18 (−0.38 to 0.02)	−0.03 (−0.21 to 0.15)
3-month follow-up	−0.01 (−0.17 to 0.15)	−0.29 (−0.49 to −0.09)	−0.13 (−0.31 to 0.06)
**Chat**			
Baseline			−0.16 (−0.34 to 0.03)
Post-intervention	−0.05 (−0.21 to 0.11)	0.03 (−0.18 to 0.23)	−0.07 (−0.25 to 0.11)
3-month follow-up	−0.12 (−0.28 to 0.04)	0.14 (−0.06 to 0.34)	0.11 (−0.07 to 0.30)
**Total non-drug treatment**			
Baseline			−0.08 (−0.26 to 0.10)
Post-intervention	−0.05 (−0.21 to 0.11)	−0.14 (−0.34 to 0.06)	−0.15 (−0.33 to 0.04)
3-month follow-up	−0.18 (−0.34 to −0.02)	−0.02 (−0.22 to 0.19)	0.09 (−0.09 to 0.27)

*PSEQ, Pain Self-Efficacy Questionnaire; BPI, Brief Pain Inventory; GDS, Geriatric Depression Scale*.

### Pain Intensity and Pain Interference

The GEE analyses revealed significant Time X Group interactions were observed on the BPI-Interference (Wald's χ^2^ = 6.50, *P* = 0.039), but not on BPI-Intensity (Wald's χ^2^ = 2.58, *P* = 0.275). On the other hands, there is significant main effect of time on BPI-Intensity (Wald's χ^2^ = 6.39, *p* = 0.041). This indicated that both experimental group and control have a significant reduce on BPI-Intensity in 3-month-follow-up (*p* = 0.022).

Therefore, planned contrasts would only be performed on BPI-Interference. Within-group comparison revealed statistically significant reductions from baseline to post-intervention on BPI-Interference (*p* = 0.001) for the experimental group, while no significant difference was found for the control group (*p* = 0.676). Importantly, at post-intervention, between-group comparison revealed that the experimental group had lower scores compared to the control group on the PSEQ (*p* = 0.009). In terms of follow-up, for the experimental group, there were no differences between post-intervention and 3-month follow-up scores for the experimental group (*p* = 0.351) and the control group (*p* = 0.368). This indicate that the improvement in post-intervention would be sustain in 3-month follow-up.

Within- and between-group effect sizes from the GEE models are shown in Table 4. Relative to the baseline, small within-group effect sizes (Cohen's d) were observed for the BPI-Interference at post-intervention (*d* = 0.15) and 3-month follow-up (*d* = 0.20) for experimental group compared to minimal effect sizes for control group for the BPI-Interference at post-intervention (*d* = −0.02) and 3-month follow-up (*d* = 0.05). For the experimental group relative to the control group, small between-group effect sizes (Cohen's d) were observed for the post-intervention (*d* = −0.18) and 3-month follow-up (*d* = −0.13).

### Perceived Quality of Life and Depression

The GEE analyses revealed significant Time X Group interactions were observed on the GDS (Wald's χ^2^ = 10.504, *P* = 0.005), but not on SF-12 Physical (Wald's χ^2^ = 0.092, *P* = 0.955) and SF-12 Mental (Wald's χ^2^ = 0.872, *P* = 0.646). Also, no significant main effect of time was found on SF-12 Physical (Wald's χ^2^ = 5.16, *P* = 0.076) and SF-12 Mental (Wald's χ^2^ = 1.36, *p* = 0.506). Therefore, planned contrasts would only be performed on GDS. Within-group comparison revealed statistically significant increases from baseline to post-intervention on GDS (*p* = 0.007) for the control group, while no significant difference was found for the experimental group (*p* = 0.226). Importantly, at 3-month follow-up, between-group comparison revealed that the experimental group had lower scores compared to the control group on the GDS (*p* = 0.025). In terms of follow-up, there were no differences between post-intervention and 3-month follow-up scores for the experimental group (*p* = 0.535) and the control group (*p* = 0.123). This indicate that the improvement in post-intervention would be sustain in 3-month follow-up.

Within- and between-group effect sizes from the GEE models are shown in Table 4. Relative to the baseline, small within-group effect sizes (Cohen's d) were observed for the GDS at post-intervention (*d* = −0.18) and 3-month follow-up (*d* = −0.29) for control group compared to minimal effect sizes for experimental group for the GDS at post-intervention (*d* = −0.05) and 3-month follow-up (*d* = −0.01). For the experimental group relative to the control group, minimal between-group effect sizes (Cohen's d) were observed for the post-intervention (*d* = −0.03) and small between-group effect sizes for 3-month follow-up (*d* = −0.13).

### Non-drug Treatments

The GEE analyses revealed significant Time X Group interactions were observed on the chat (Wald's χ^2^ = 6.214, *p* = 0.005) and total non-drug treatment only (Wald's χ^2^ = 12.121, *p* = 0.026). On the other hand, significant main effect of time was only found on massage (Wald's χ^2^ = 7.83, *p* = 0.020), rest (Wald's χ^2^ = 15.59, *p* < 0.001), watch TV (Wald's χ^2^ = 11.70, *p* = 0.003), breath (Wald's χ^2^ = 6.12, *p* = 0.047), and exercise (Wald's χ^2^ = 16.81, *p* < 0.001). This indicated that both experimental group and control have a significant reduced on rest and increased on watch TV, breath, and exercise in 3-month-follow-up (*p*-range: <0.001–0.025), while massage only have a significant increased on post-intervention only (*p* = 0.006).

Therefore, planned contrasts would only be performed on chat and total non-drug treatment. Within-group comparison revealed statistically significant increases from baseline to 3-month follow-up on total non-drug treatment (*p* = 0.009) for the experimental group, while no significant difference was found for the control group (*p* = 0.144). On the other hands, no significant differences were found for within-group comparison, and between-group comparison in post-intervention and 3-month follow-up for chat.

Within- and between-group effect sizes from the GEE models are shown in Table 4. Relative to the baseline, small within-group effect sizes (Cohen's d) were observed for the total non-drug treatment at 3-month follow-up (*d* = −0.18) for experimental group compared to minimal effect sizes for control group 3-month follow-up (*d* = −0.02). While small within-group effect sizes (Cohen's d) were observed for the chat at 3-month follow-up for both experimental group (*d* = −0.12) and control group at 3-month follow-up (*d* = 0.14).

### Pain Knowledge

The GEE analyses revealed that there was no significant Time X Group interactions were found on the pain knowledge (Wald's χ^2^ = 0.44, *P* = 0.801). On the other hands, there are significant main effect of time on pain knowledge (Wald's χ^2^ = 9.82, *P* = 0.007). This indicated that both experimental group and control have a significant increase on pain knowledge in post-intervention (*p* = 0.013) and 3-month-follow-up (0.009).

## Discussion

The present study used a peer support models and proven to be effective in managing pain and pain related situations for nursing home residents. Our clustered randomized controlled trial examined the effectiveness of peer-led pain management program (PAP). Our results add insight to the pain management situation of nursing home residents.

There were 262 participants joined the study. Before our intervention, the mean pain score reported in the present study was as high as 6.36 when scored on a 10-point Likert Scale. The high intensity of their pain very much interfered with the daily activities of the participants. Their ability to walk, for example, was restricted. Pain interference was high and the participants had poor coping as indicated by the low pain self-efficacy. Depression and a low quality of life score was observed. Upon completion of our PAP, there was a significant increase in pain self-efficacy, pain interference for the participants in the experimental group and not in the control group, and this improvement sustained in 3-month follow up.

The present study used a peer support model. Being a peer volunteer is a good way to continue contributing to the society (13, 19). Volunteering activities improve the emotions and feelings of the volunteers, of which, give sense of satisfaction in life (20). Indeed, the health benefits of being volunteers include increase in physical activities, improvements in self-reported health and well-being, more satisfied in life, reduced in pain and depressive moods and symptoms (19, 20). Peer volunteers are not constrained by time and are readily available. Drawing upon this resource can help to save on healthcare resources (13, 19). It is observed that once our peer volunteers are trained, they can be empowered and remain involved in the program and serving the older adults in the nursing homes.

Populations are aging worldwide; including the Hong Kong population (21), as well as the elderly people living in nursing homes is also increasing (22). Indeed, more than 40% of the participants in our study had been living in a nursing home for more than 3 years, and nearly 6% of them had resided in the nursing home for over 10 years. Our results also showed severe pain intensity and low self-efficacy among the participants, which warranted prompt actions. Our intervention, the PAP tapped on the resources and the benefits of using peers, and equipped them with knowledge and training, to lead pain management program for nursing home older adults, with promising results.

Pain self-efficacy refers to a patient's belief in his or her ability to accomplish daily tasks in spite of pain (15). The low pain self-efficacy reflects the participants' lack of belief in their ability to deal with pain. Pain interference refers to the extent to which the pain interferes with activities such as repositioning, deep breathing/coughing, walking, mood swings, chatting, and sleeping (17). In the present study, pain self-efficacy improved and pain interference reduced over time for the participants in the experimental group, but not for the control group. In this way, upon completion of the PAP, older adults were more capable of handling their daily tasks and pain becomes less disturbing/interfering their daily activities, of which, they are more able to enjoy their daily lives.

In our study, the baseline data of the quality of life was much lower than that found in a study conducted in 2013 involving over 2,000 participants from the general population (23). Upon completion of the PAP, the quality of life scores for participants in the experimental group and control group had no significant difference; yet, the geriatric depression score had been increasing in the control group, indicating they felt more depressed over time as compared to the experimental group.

There has been an increased in the use of non-drugs treatment for the participants in our study. A systematic review from Tang et al. (24). suggests that non-pharmacological interventions are suitable and sustainable in pain relief. Indeed, non-pharmacological interventions are effective and safe in reducing pain intensity with fewer side effects and adverse outcomes as compared with pharmacological interventions (25). It is important to provide pain management education, advocating the use of non-drugs treatment, to older adults, and to those living in nursing homes in particular. Given the high prevalence and high intensity of pain situations among nursing home residents, who are already physically frail, live in “closed” nursing home environments, and may have difficulty seeking pain management strategies.

As for pain knowledge, although there are no interventional effecct, there has been an increased in post-intervention and 3-month follow-up for the participants in our study, no matter it is experimental group or control group. The score for the participants was 47.49 out of 100, and the score was increased to 51.98 and 53.45 in post-intervention and 3-month follow-up respectively. A higher score indicates more knowledge. Insufficient knowledge about pain may lead to low pain self-efficacy, which is consistent with the low pain self-efficacy found in our study.

There are several limitations in this study. Because the data that were collected had been reported by the participants, different individuals might have understood the questions differently, which might have affected the overall results. Yet, every efforts were made by the research team, by speaking clearly, in a slow pace and reduce the background noise of the environment in order to allow participants to gain a full understanding of the questions. In addition, the participants might have provided socially desirable responses to please the researchers.

## Conclusion

The present study used a peer support models and proven to be effective in managing pain and pain related situations for nursing home residents with chronic pain. The peer volunteers involved in the pain management program taught relevant pain knowledge and pain management strategies to help our participants. A total of 262 participants with high pain scores prior to the program were successfully recruited. Pain very much interfered with their ability to walk. Upon completion of the pain management program, pain self-efficacy improved and pain inference reduced over time for experimental group and not for control group. Our results add insight to the pain management situations of nursing home residents.

## Data Availability Statement

The raw data supporting the conclusions of this article will be made available by the authors, without undue reservation.

## Ethics Statement

The studies involving human participants were reviewed and approved by The Ethics Committee of The Hong Kong Polytechnic University. The patients/participants provided their written informed consent to participate in this study.

## Author Contributions

ST, YL, and PL: data collection. PL, MT, and KC: formal analysis. MT: funding acquisition and project administration. RL: resources. MT, SN, and RL: supervision. MT, YL, KC, and ST: writing—original draft. MT, YL, XB, RL, and KC: writing—review and editing. All authors contributed to the article and approved the submitted version.

## Conflict of Interest

The authors declare that the research was conducted in the absence of any commercial or financial relationships that could be construed as a potential conflict of interest.

## Publisher's Note

All claims expressed in this article are solely those of the authors and do not necessarily represent those of their affiliated organizations, or those of the publisher, the editors and the reviewers. Any product that may be evaluated in this article, or claim that may be made by its manufacturer, is not guaranteed or endorsed by the publisher.

## Appendix

**Appendix 1:** Teaching manual for the experimental group.

**Outline of the pain management program (PAP)**			
**Content**		**Pain management program (PAP)**	
**Week**	**Physical exercise (20 min)**	**Interactive teaching and sharing of pain management education (30 min)**	**Portfolio entry (10 min)**
1	Correct body posture and alignment, Stretching of arms, legs, and body muscles; Balancing exercise; Shoulder & neck exercise; Hip exercise; Knee exercise; Towel dancing	Pain situations among themselves; Effects of pain in their daily life; Can we do something?	Peer volunteers worked with the participants to make entries on the activity of the day in the “I can do it” booklet.
2		The use of an oral drug: effects & side-effects; labels of oral drugs	
3		The use of a non-drug therapy: Hot pads & cold pads; how to use & safety issues	
4, 5		The use of a non-drug therapy: listening to music	
6, 7		The use of a non-drug therapy: Massage	
8, 9		The use of a non-drug therapy: visual stimulation – watching the natural environment, & making a photo album	
10, 11		The use of a non-drug therapy: sense of smell & taste – making a bag of dried flowers & tasting tea	
12		Revision & Wrapping up	

*The teaching manual was developed and used in the pilot study. The content was validated by the research team, a geriatric physician consultant specializing in pain, a registered physiotherapist, and an advanced practice nurse experienced in elderly care. This manual were given to PVs and all participants at the beginning of the PAP for their reference*.

**Funding.** This research was supported by the Health and Medical Research Fund of the Food and Health Bureau, Hong Kong SAR Government (Ref. 15161051).
